# Levoketoconazole in the treatment of patients with endogenous Cushing’s syndrome: a double-blind, placebo-controlled, randomized withdrawal study (LOGICS)

**DOI:** 10.1007/s11102-022-01263-7

**Published:** 2022-09-09

**Authors:** Rosario Pivonello, Sabina Zacharieva, Atanaska Elenkova, Miklós Tóth, Ilan Shimon, Antonio Stigliano, Corin Badiu, Thierry Brue, Carmen Emanuela Georgescu, Stylianos Tsagarakis, Fredric Cohen, Maria Fleseriu

**Affiliations:** 1 Università Federico II Di Napoli, Naples, Italy; 2 Medical University Sofia, Sofia, Bulgaria; 3 Semmelweis University, Budapest, Hungary; 4 Rabin Medical Center and Tel Aviv University, Tel Aviv, Israel; 5 Sant’Andrea Hospital, University of Rome “Sapienza”, Rome, Italy; 6 National Institute of Endocrinology CI Parhon and “C. Davila” University of Medicine and Pharmacy, Bucharest, Romania; 7 Aix-Marseille Université and Assistance Publique - Hôpitaux de Marseille, Hôpital de la Conception, Marseille, France; 8 Iuliu Haţieganu University of Medicine and Pharmacy, Cluj-Napoca, Romania; 9 Endocrinology Clinical Unit, Cluj County Emergency Hospital, Cluj-Napoca, Romania; 10 Evangelismos Hospital, Athens, Greece; 11 Xeris Biopharma, Chicago, IL USA; 12 Oregon Health and Science University, Portland, OR USA

**Keywords:** Cushing’s syndrome, Cushing’s disease, Hypercortisolism, Levoketoconazole, Placebo, Steroidogenesis inhibitor

## Abstract

**Purpose:**

The efficacy of levoketoconazole for endogenous Cushing’s syndrome was demonstrated in a phase 3, open-label study (SONICS). This study (LOGICS) evaluated drug-specificity of cortisol normalization.

**Methods:**

LOGICS was a phase 3, placebo-controlled, randomized-withdrawal study with open-label titration-maintenance (14–19 weeks) followed by double-blind, randomized-withdrawal (~ 8 weeks), and restoration (~ 8 weeks) phases.

**Results:**

79 patients received levoketoconazole during titration-maintenance; 39 patients on a stable dose (~ 4 weeks or more) proceeded to randomization. These and 5 SONICS completers who did not require dose titration were randomized to levoketoconazole (n = 22) or placebo (n = 22). All patients with loss of response (the primary endpoint) met the prespecified criterion of mean urinary free cortisol (mUFC) > 1.5 × upper limit of normal. During randomized-withdrawal, 21 patients withdrawn to placebo (95.5%) lost mUFC response compared with 9 patients continuing levoketoconazole (40.9%); treatment difference: − 54.5% (95% CI − 75.7, − 27.4; *P* = 0.0002). At the end of randomized-withdrawal, mUFC normalization was observed among 11 (50.0%) patients receiving levoketoconazole and 1 (4.5%) receiving placebo; treatment difference: 45.5% (95% CI 19.2, 67.9; *P* = 0.0015). Restoration of levoketoconazole reversed loss of cortisol control in most patients who had received placebo. Adverse events were reported in 89% of patients during treatment with levoketoconazole (dose-titration, randomized-withdrawal, and restoration phases combined), most commonly nausea (29%) and hypokalemia (26%). Prespecified adverse events of special interest with levoketoconazole were liver-related (10.7%), QT interval prolongation (10.7%), and adrenal insufficiency (9.5%).

**Conclusions:**

Levoketoconazole reversibly normalized urinary cortisol in patients with Cushing’s syndrome. No new risks of levoketoconazole treatment were identified.

**Supplementary Information:**

The online version contains supplementary material available at 10.1007/s11102-022-01263-7.

## Introduction

Endogenous Cushing’s syndrome (CS) is a rare endocrine disease caused by chronic overproduction of cortisol, most commonly due to a benign pituitary corticotroph tumor (Cushing’s disease [CD]) [1]. Patients with endogenous CS have multiple associated comorbidities [2–4] and increased mortality risk, primarily as a result of cardiovascular complications and infections [2, 5–8]. Surgery to remove the inciting tumor is usually the first-line treatment for CS of pituitary (CD), adrenal, or ectopic etiologies [9–11]. However, persistent or recurrent disease after initial pituitary surgery is common [12–14], requiring second-line treatments in patients with CD [9–11]. Medical therapy may be used prior to surgery, while awaiting the effects of radiation therapy, when surgery is contraindicated or when a tumor cannot be found, and when biochemical remission is not achieved with surgery and/or radiation [9–11, 15]. Available medical therapies for CS vary by mechanism of action and include pituitary-directed drugs (for CD; pasireotide, cabergoline), cortisol synthesis inhibitors (levoketoconazole, osilodrostat, ketoconazole, metyrapone, mitotane [adrenolytic]), and a glucocorticoid receptor antagonist (mifepristone) [10, 16, 17]. It is generally agreed that medical needs in CS remain high despite the availability of approved drug treatments.

Ketoconazole, a racemic mixture of two enantiomers (levoketoconazole [*2S,4R*-ketoconazole] and dextroketoconazole [*2R,4S*-ketoconazole]), is not approved by the US Food and Drug Administration for use in patients with CS, but off-label use is common [17, 18]. Ketoconazole was approved by the European Medicines Agency for the treatment of CS on the basis of published information, including clinical data collected in observational studies; no prospective interventional clinical studies were available [19]. Ketoconazole reduces adrenal cortisol production by inhibiting several adrenal steroidogenic enzymes [18, 20]. Research suggests that most of the cortisol suppression observed after ketoconazole administration appears to be inherent to levoketoconazole [21–23].

A phase 3, open-label study in patients with CS (SONICS) demonstrated the efficacy of levoketoconazole in normalizing cortisol levels during the dose titration phase, with durability of response demonstrated during a 6-month maintenance phase [24–26]. Of 94 patients who entered the SONICS study, mUFC was normalized at the end of the maintenance phase (without a dose increase during maintenance) in 30% of patients (primary endpoint; 95% confidence interval (CI) 21%–40%; *P* = 0.0154 vs. null hypothesis of ≤ 20%). Among 55 patients who completed the maintenance phase, mUFC was normalized in 34 patients (62%) and ≥ 50% mUFC decrease or normalization was observed in 43 patients (78%), irrespective of dose increase. The current study (LOGICS) further evaluated the safety and efficacy of levoketoconazole in patients with CS, with the primary objective of determining the effect of withdrawing levoketoconazole treatment to placebo versus continuing treatment with levoketoconazole on the cortisol therapeutic response achieved during open-label levoketoconazole therapy, in order to establish efficacy as specifically related to levoketoconazole.

## Methods

### Study design and patients

**Fig. 1:**
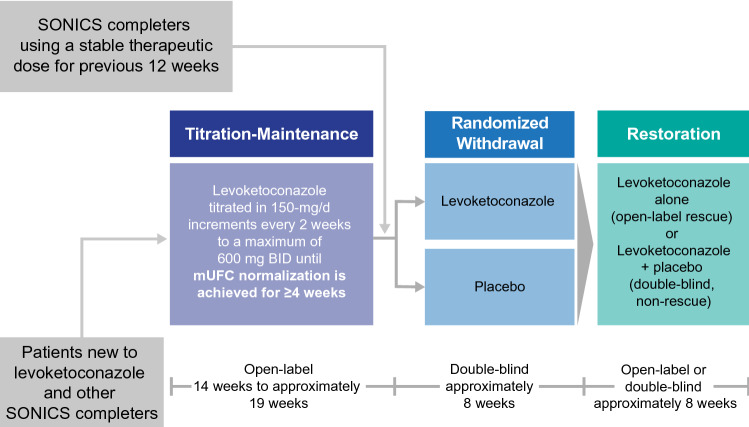
Study design. *BID* twice daily, *mUFC* mean urinary free cortisol

LOGICS was a randomized, double-blind, placebo-controlled withdrawal and restoration (or, as needed, early rescue) study in patients with endogenous CS. The study included an open-label titration-maintenance (TM) phase to establish the therapeutic dose for each patient, a randomized withdrawal (RW) phase to evaluate efficacy in a double-blind comparison to placebo, and a restoration phase to re-establish treatment with the previously established therapeutic dose of levoketoconazole (Fig. 1). The study was conducted in accordance with the ethical principles set forth in the Declaration of Helsinki and the Good Clinical Practice guidelines of the International Council on Harmonisation. The study protocol was approved by an independent ethics committee or institutional review board at each study site. All patients provided written informed consent to participate.

Patients were enrolled at 36 sites in 11 countries (9 European countries, Israel, and the United States). Key inclusion criteria were: age 18 years or more; confirmed newly diagnosed, persistent, or recurrent endogenous CS of any etiology except CS secondary to malignancy (including pituitary or adrenal carcinoma); 24-h mean urinary free cortisol (mUFC) levels at least 1.5 × the upper limit of normal (ULN) with no upper bounds, based on at least 3 measurements from adequately collected urine; abnormal dexamethasone suppression test and/or elevated late-night salivary cortisol (LNSC) concentration; and not being a candidate for CS-specific surgery during the study.^1^ Key exclusion criteria included: enrollment in SONICS without completion through 12 months of maintenance treatment; suspected pseudo-CS, cyclic CS, or a nonendogenous source of hypercortisolism; radiotherapy within the previous 5 years; pituitary surgery within the previous 6 weeks; history of adrenal or pituitary carcinoma; compression of the optic chiasm; QT prolongation or abnormal electrocardiogram (ECG) requiring medical intervention; history of torsade de pointes, ventricular tachycardia, ventricular fibrillation, or long QT-syndrome (including first-degree family history); pre-existing hepatic disease (apart from mild to moderate nonalcoholic fatty liver disease); alanine aminotransferase (ALT) or aspartate aminotransferase (AST) > 3 × ULN; alkaline phosphatase or total bilirubin > 2 × ULN; persistent, uncontrolled hypertension; and poorly controlled diabetes.

### Procedures

Eligible SONICS study completers on a stable therapeutic dose of levoketoconazole were directly randomized. The remaining enrolled patients entered the TM phase. Open-label levoketoconazole was started at an initial dose of 150 mg (one oral tablet) twice daily; SONICS completers re-establishing a therapeutic dose via re-titration could start titration at their current or most recently received dose at the discretion of the investigator. Individualized levoketoconazole dosing was established by titration in increments of 150 mg/day every 2 weeks, as needed to achieve mUFC normalization, until a therapeutic dose (defined as the dose providing mUFC at or below the ULN, determined from 3 adequate 24-h urine collections) was established, at which point the dose regimen was maintained unless further adjustment was required. A urine sample was considered to be adequate for analysis purposes if the creatinine excretion rate was 4.5 mg/kg/day or greater for females and 6.2 mg/kg/day or greater for males, these thresholds representing 2 standard deviations below the mean of pooled samples in a prior study (Study COR-2012-01, data on file). UFC was assayed at a central laboratory by high-performance liquid chromatography–tandem mass spectroscopy (ULN of 138 nmol/24 h). Patients were eligible to enter the RW phase if they established a therapeutic dose prior to the end of TM phase, continued receiving the dose associated with mUFC normalization for at least the final 4 weeks in TM phase, and completed the final visit of TM phase.

Eligible patients from the TM phase and SONICS completers who did not require re-titration were randomly assigned in a 1:1 ratio to receive either blinded levoketoconazole at the therapeutic dose, or the same regimen with matching placebo tablets, for up to 8 weeks without dose adjustment. Early rescue during the RW phase was allowed when a patient demonstrated relapse of hypercortisolemia (i.e., loss of therapeutic response), which was defined as (1) mUFC (from three 24-h urine collections) above 1.5 × ULN or (2) for SONICS completers with mUFC above the ULN at baseline, an increase in mUFC more than 40% above the baseline value. If early rescue was needed, patients immediately entered the restoration phase, and open-label levoketoconazole was rapidly titrated (dose increased in 1-tablet increments every 2 days, alternating morning and evening) to the therapeutic dose. The treatment blind was maintained in the restoration phase for nonrescued patients by addition of the alternative treatment regimen to the randomized regimen (i.e., masked placebo tablets added to levoketoconazole regimen or vice versa), such that all patients received levoketoconazole during the restoration phase.

### Outcome measures

The primary endpoint was the proportion of patients with loss of therapeutic response to levoketoconazole upon withdrawing to placebo, compared with the proportion of patients with loss of therapeutic response upon continuing treatment with levoketoconazole. Loss of therapeutic response was inferred based on mUFC from three 24-h urinary free cortisol (UFC) measurements obtained at any of 5 visits during the RW phase and was defined using the criteria for early rescue described above. The key secondary endpoint was the proportion of patients with normalization of mUFC at the end of the RW phase.

Additional secondary endpoints included change from RW baseline to end of RW phase for mUFC, biomarkers of CS comorbidities (e.g., fasting glucose, fasting insulin, homeostatic model assessment-insulin resistance [HOMA-IR], HbA1c, total cholesterol, LDL-cholesterol, high-sensitivity C-reactive protein [hsCRP]), health-related QoL, symptoms of depression, physical signs of CS (i.e., acne, hirsutism, peripheral edema), and LNSC. mUFC was measured from 2 (most assessments) or 3 (to determine eligibility, therapeutic dose, and need for early rescue; and at final RW phase and final restoration phase assessments) adequate 24-h urine specimens that were collected on consecutive days. Biomarkers of CS comorbidities were measured from blood samples using standard clinical laboratory assays. Health-related QoL was assessed using the CushingQoL questionnaire [27], and the severity of depression was assessed using the Beck Depression Inventory-II (BDI-II) [28]. Acne [29], hirsutism (females only) [30], and peripheral edema [31] were evaluated by the investigator using specific severity grading systems. LNSC was measured from 1 or 2 (consecutive night) saliva collections performed between 11 pm and midnight.

Safety assessments included adverse event (AE) monitoring, clinical laboratory tests, vital sign measurement, and ECG. AEs were coded in accordance with the Medical Dictionary for Regulatory Activities (MedDRA) version 21.1. AEs considered to be of special interest (i.e., liver-related, QT interval–related, adrenal insufficiency) were immediately reported by the investigator (and did not necessarily include all recorded AEs for the associated MedDRA preferred terms). Additional safety monitoring in patients with CD included plasma adrenocorticotropic hormone (ACTH) levels and pituitary magnetic resonance imaging (MRI; for tumor size measurement).

### Statistical analysis

A sample size of 44 patients (22 per treatment group) completing the RW phase was estimated to provide approximately 98% power, using two-sided Fisher’s exact test, to detect the alternative hypothesis of a loss of therapeutic response rate of 26% in the levoketoconazole group and 81% in the placebo group (i.e., a treatment difference of −55%) versus the null hypothesis of no difference between groups.

The primary efficacy analysis (loss of therapeutic response during the RW phase) was conducted using a logistic regression model containing fixed-effect terms for treatment group and patient cohort (TM phase completers versus those directly randomized from SONICS). A prespecified supportive analysis compared the proportions of patients with loss of response using a 2-sided Fisher’s exact test. Results from the Fisher’s exact test are considered as the primary outcome because imbalance in regression model fixed effects inflated the apparent treatment effect of levoketoconazole. Sensitivity analyses evaluated variations in the analysis procedures including (1) single adequate urine sample allowed for calculation of mUFC and imputation of missing mUFC using the last observation carried forward (LOCF), (2) calculation of mUFC using all samples, regardless of adequacy, and (3) at least 2 postrandomization RW phase visits with adequate mUFC to determine loss of response.

Inferences derived from secondary efficacy analyses were gated on results from the primary efficacy analysis to ensure control of the familywise type I error rate at 0.05. Secondary efficacy analyses were tested sequentially in 6 sets as follows: (1) normalization of mUFC at the end of the RW phase, followed by changes from RW baseline to end of RW in: (2) mUFC, (3) biomarkers of CS comorbidities, (4) CushingQoL and BDI-II total scores, (5) acne global score and hirsutism and peripheral edema total scores; and (6) LNSC. The proportions of patients with normalization of mUFC at the end of the RW phase were compared between treatment groups using the Fisher’s exact test. For all other secondary endpoints, mean changes from RW phase baseline to the end of the RW phase were compared between treatment groups using 2-sample t-tests.

## Results

### Patients

**Fig. 2:**
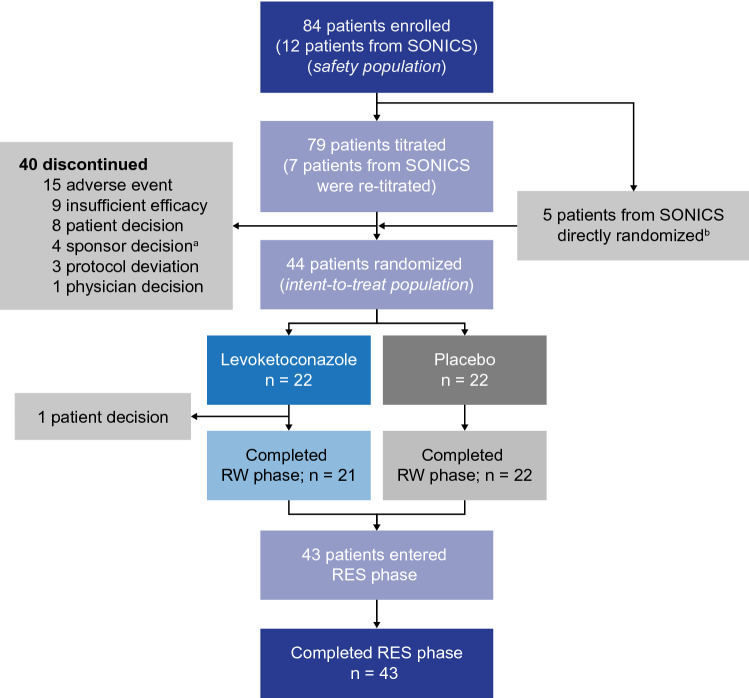
Patient disposition. ^a^Sponsor decision to halt randomization; these patients were enrolled into the long-term open-label extension study (OPTICS). ^b^5 patients who directly rolled over to LOGICS (levoketoconazole: n = 1; placebo: n = 4) from SONICS were on a stable therapeutic dose for 12 weeks prior to screening and did not require dose titration. *RES* restoration, *RW* randomized withdrawal

The safety population comprised 84 patients who received levoketoconazole at any time during the study (Fig. 2). The intent-to-treat (ITT) population included 44 patients (levoketoconazole, n = 22; placebo, n = 22) who received study medication in the RW phase. Combining all phases, 41 of the 84 patients in the safety population discontinued the study prematurely, most commonly due to AEs (n = 15), insufficient efficacy (n = 9), and patient decision (n = 9).

Demographic and baseline characteristics were similar for the safety and ITT populations (Table 1). In the ITT population, median time since diagnosis was shorter in the levoketoconazole group versus the placebo group (36 vs. 82 months), mean mUFC at study baseline was higher (5.4 × ULN vs. 3.0 × ULN), and prior diagnosis of hypertension was more frequent (95% vs. 73%). Among the 70 enrolled patients with CD, 47 (67%) had at least one prior pituitary surgery.

**Table 1:** LOGICS study: demographics and baseline characteristics

Characteristics	Safety population (n = 84)	Intent-to-treat population	
		Levoketoconazole (n = 22)	Placebo (n = 22)
Age, years, mean (SD)	44.7 (12.7)	45.0 (12.0)	43.6 (11.0)
Female, n (%)	64 (76.2)	15 (68.2)	19 (86.4)
Race, n (%)			
White	78 (92.9)	18 (81.8)	22 (100)
Black	4 (4.8)	3 (13.6)	0 (0)
Asian	1 (1.2)	0 (0)	0 (0)
Unknown	1 (1.2)	1 (4.5)	0 (0)
BMI, kg/m^2^, mean (SD)	31.0 (6.8)	31.6 (8.5)	30.8 (4.8)
Time since CS diagnosis, months			
Mean (SD)	63.4 (71.8)	66.8 (72.5)	92.2 (78.8)
Median (range)	30.1 (0–254.1)	35.8 (0.5–241.0)	82.1 (0.2–254.1)
Etiology, n (%)			
Cushing’s disease	70 (83.3)	18 (81.8)	20 (90.9)
Adrenal-dependent	8 (9.5)	3 (13.6)	1 (4.5)
Ectopic ACTH secretion	2 (2.4)	0 (0)	0 (0)
Unknown	4 (4.8)	1 (4.5)	1 (4.5)
Diabetes, n (%)	35 (41.7)	8 (36.4)	7 (31.8)
Hypertension, n (%)	68 (81.0)	21 (95.5)	16 (72.7)
Baseline mUFC, nmol/24 h^a^			
Mean (SD)	746.7 (916.3)	738.7 (1067.0)	411.6 (436.2)
Median (range)	441.6 (53.1–5752.9)	382.9 (101.9–5004.9)	266.8 (53.1–2171.3)
Baseline mUFC, × ULN^a,b^			
Mean (SD)	5.4 (6.6)	5.4 (7.7)	3.0 (3.2)
Median (range)	3.2 (0.4–41.7)^c^	2.8 (0.7–36.3)^c^	1.9 (0.4–15.7)^c^

*ACTH* adrenocorticotropic hormone, *BMI* body mass index, *CS* Cushing’s syndrome, *mUFC* mean urinary free cortisol, *SD* standard deviation, *UFC* urinary free cortisol, *ULN* upper limit of normal

^a^For each patient, the average of the UFCs from adequate samples at baseline was calculated

^b^ULN for UFC = 138 nmol/24 h

^c^Patients with mUFC < ULN directly entered from SONICS

### Efficacy

#### Cortisol control

**Fig. 3:**
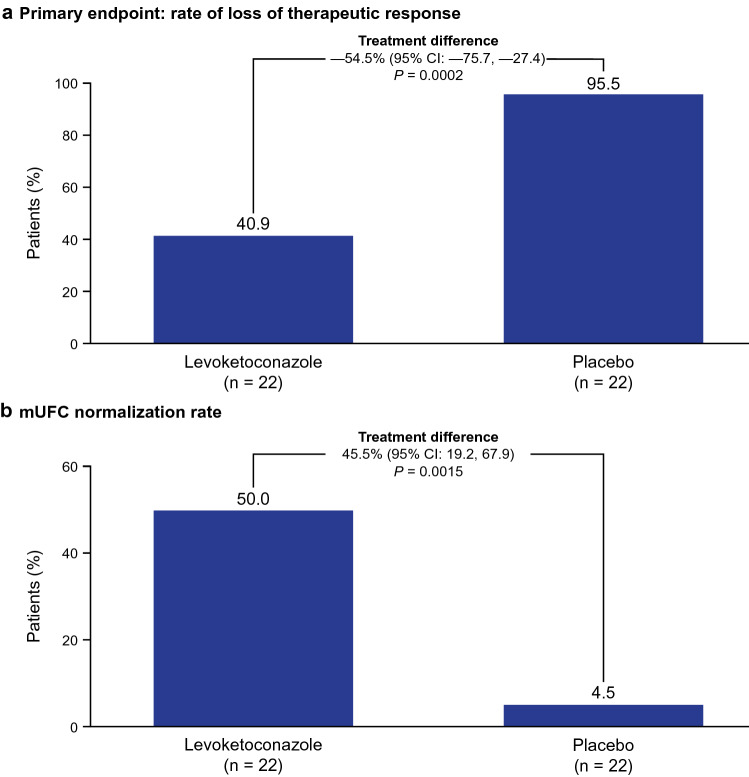
Proportion of patients who met **a** the primary endpoint, loss of therapeutic response during the RW phase, or **b** the key secondary endpoint, mUFC normalization at the end of the RW phase (ITT population). Loss of therapeutic response defined as mUFC > 1.5 × ULN (or mUFC > 40% above baseline if RW baseline was > 1.0 × ULN), or other rescue criterion met. 2 patients from the group that entered RW from TM phase had mUFC above ULN at RW baseline (1 in each treatment group). 4 patients in the levoketoconazole group and 21 patients in the placebo group received early rescue therapy. *ITT* intent-to-treat, *mUFC* mean urinary free cortisol, *RW* randomized-withdrawal, *TM* titration-maintenance, *ULN* upper limit of normal

During the RW phase, 21 of 22 patients receiving placebo (95.5%) demonstrated loss of mUFC response compared with 9 of 22 patients receiving levoketoconazole (40.9%); the treatment difference estimate of –54.5% significantly favored levoketoconazole (95% CI − 75.7, − 27.4; *P* = 0.0002; Fig. 3A). Early rescue therapy was provided to 4 (18%) of 22 patients in the levoketoconazole group and 21 (95%) of 22 patients in the placebo group, all of whom had relapse of hypercortisolemia based on mUFC greater than 1.5 × ULN. Five additional patients in the levoketoconazole group lost response (i.e., mUFC greater than 1.5 × ULN); however, they did not receive early rescue treatment because 4 patients had loss of response at the last RW visit and 1 patient had loss of response at the first RW visit (based on 2 specimens) but mUFC was < 1.5 × ULN at all subsequent RW visits. At the end of the RW phase, normalization of mUFC was observed among 11 (50.0%) patients in the levoketoconazole group compared with 1 (4.5%) in the placebo group; the treatment difference estimate of 45.5% favored levoketoconazole (95% CI 19.2, 67.9; *P* = 0.0015; Fig. 3B). Multiple sensitivity analyses of both the primary and key secondary efficacy endpoints corroborated the main analytic inferences. The time to loss of therapeutic response or early rescue was significantly shorter for the placebo group, with a Kaplan–Meier estimated median time of 24 days (95% CI 19, 31) compared with 62 days (95% CI 40, NA [not calculable]) for the levoketoconazole group (log-rank *P* < 0.0001; Online Resource 1).

**Fig. 4:**
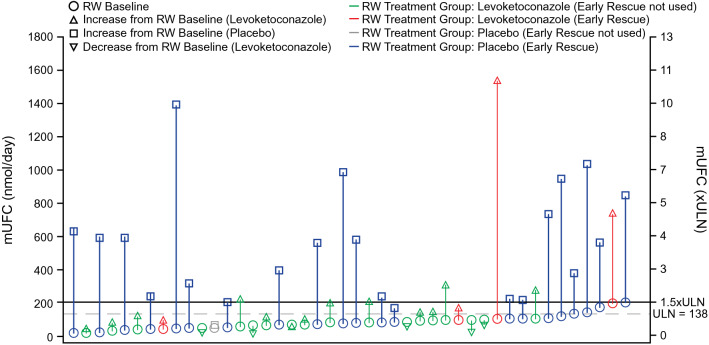
Changes in individual mUFC concentrations from RW baseline through the end of the RW (ITT population). Plot displays individual patient mUFC from RW baseline to the end of the RW phase. Each needle begins at RW baseline value (last nonmissing result prior to the first dose of study drug administration during the RW phase) and ends at the last measurement of mUFC in the RW phase (before early rescue in cases where early rescue occurred). *ITT* intent to treat, *mUFC* mean urinary free cortisol, *RW* randomized withdrawal, *ULN* upper limit of normal

During the TM phase, mean (SD) mUFC decreased from 780.8 (936.4) nmol/24 h at baseline to 304.2 (638.9) nmol/24 h for the last observed value (n = 77). mUFC normalization rates during the TM phase, by levoketoconazole dose, are shown in Online Resource 2. Higher TM baseline mUFC generally was associated with higher doses of levoketoconazole used at the time of the last TM mUFC assessment. Among patients who entered the RW phase, mean (SD) mUFC for patients randomized to levoketoconazole was 81.3 (35.7) nmol/24 h at RW phase baseline, increasing by a mean of 139.4 nmol/24 h to 220.8 (333.5) nmol/24 h at the end of the RW phase. In the placebo group, baseline mean (SD) mUFC was 88.4 (48.1) nmol/24 h, increasing by a mean of 453.5 nmol/24 h to 541.9 (341.4) nmol/24 h at the end of the RW phase (treatment difference − 314.0 nmol/24 h favoring levoketoconazole, *P* = 0.0027). Examination of individual patient trajectories in mUFC over the duration of the RW phase illustrates the need for rescue treatment, predominantly in the placebo group (Fig. 4).

#### Comorbidity biomarkers

During the TM phase, mean reductions from baseline were observed for total cholesterol, LDL-cholesterol, fasting glucose, HbA1c, fasting insulin, and HOMA-IR (Table 2). The mean RW phase baseline values for each of the CS biomarkers were within the normal range for both treatment groups, with the exception of fasting insulin and hsCRP, which were above the normal range for both groups and the placebo group, respectively. Statistically significant treatment group differences in mean changes from RW baseline were observed only for total and LDL-cholesterol, both favoring levoketoconazole (Table 2). Because some, but not all, CS biomarkers exhibited a statistically significant difference between treatment groups, analyses of all remaining secondary endpoints were considered exploratory.

**Table 2:** Mean changes in biomarkers of CS comorbidities in the titration maintenance and randomized withdrawal phases

Biomarker	TM phase		RW phase Baseline mean (SD)		Change from RW baseline at the end of the RW phase Mean (SD)			
	Levoketoconazole (n = 79)							
	Baseline mean (SD)	Mean (SD) change	Levoketoconazole (n = 22)	Placebo (n = 22)	Levoketoconazole (n = 22)	Placebo (n = 22)	Treatment difference^a^	Adjusted *P* value^b^
LDL-cholesterol (mmol/L)	2.8 (0.9)	− 0.6 (0.9)	2.0 (0.6)	2.0 (0.8)	− 0.006 (0.6)	0.6 (0.6)	− 0.7 (0.2)	0.0056
Total cholesterol (mmol/L)	5.3 (1.2)	− 0.7 (1.1)	4.1 (0.8)	4.3 (0.8)	− 0.04 (0.6)	0.9 (0.8)	− 1.0 (0.2)	0.0004
FBG (mmol/L)	5.9 (1.8)	− 0.4 (1.1)	5.2 (0.7)	5.3 (1.2)	− 0.09 (0.6)	0.05 (0.6)	− 0.1 (0.2)	0.4535
HbA1c (%)	6.2 (1.4)	− 0.5 (0.7)	5.6 (0.7)	5.6 (0.6)	− 0.06 (0.3)	0.08 (0.2)	− 0.1 (0.1)	0.2856
hsCRP (mg/L)	3.9 (9.3)	0.3 (7.6)^c^	2.8 (6.3)	6.4 (19.2)	1.3 (3.2)	− 4.8 (19.4)	6.1 (4.2)	0.4535
Fasting insulin (pmol/L)	116.8 (55.0)	− 4.3 (49.7)	127.6 (107.3)	100.3 (88.1)	− 14.5 (95.5)	9.3 (57.4)	− 23.8 (25.4)	0.4535
HOMA-IR	4.9 (2.7)	− 0.4 (2.8)	5.0 (4.9)	4.0 (4.2)	− 0.7 (4.6)	0.3 (3.0)	− 1.0 (1.2)	0.4535

Sample sizes vary slightly across specific measurements due to data availability

Reference ranges: mUFC: ULN = 138 nmol/24 h; LDL-cholesterol: 0–3.35 mmol/L; total cholesterol: 0–5.15 mmol/L; FBG: 3.9–6.4 mmol/L (13–49 years old), 3.9–6.9 mmol/L (≥ 50 years old); HbA1c: 0–6.5%; hsCRP: 0–3 mg/L; fasting insulin: 30–90 pmol/L; HOMA-IR: reference range not established

*CS* Cushing’s syndrome, *FBG* fasting blood glucose, *HbA1c* hemoglobin A1c, *HOMA-IR* homeostatic model assessment of insulin resistance, *hsCRP* high-sensitivity C-reactive protein, *LDL* low-density lipoprotein, *RW* randomized withdrawal, *SD* standard deviation, *TM* titration maintenance

^a^Values shown are mean (SD) except for treatment difference, which is mean (standard error)

^b^Treatment comparison at the end of the RW phase by two-sample t-test with Hochberg adjustment

^c^With outlier removed

#### Quality of life, depression, and signs and symptoms of Cushing’s syndrome

At RW baseline, mean CushingQoL scores were consistent with moderate QoL impairment on average, and mean BDI-II scores were indicative of minimal to mild depression. Mean score changes from RW baseline to the end of the RW phase for CushingQoL, BDI-II, and physical signs of CS (i.e., acne, hirsutism in females, peripheral edema) numerically favored levoketoconazole; however, none of the treatment group comparisons were statistically significant (Online Resource 3).

#### Late-night salivary cortisol

At RW baseline, mean (SD) LNSC concentrations were 6.0 (5.3) nmol/L and 6.4 (5.4) nmol/L in the levoketoconazole and placebo groups, respectively. Mean LNSC concentrations increased at the end of the RW phase in both groups by 2.2 and 2.6 nmol/L, respectively (calculated without 2 outliers, 1 in each treatment arm). The treatment difference in mean change during the RW phase (− 0.5) was not statistically significant.

#### Restoration phase

During the restoration phase, all patients received active treatment with levoketoconazole. Normalization of mUFC at the end of the restoration phase was reported among 11 (52%) of 21 patients in the prior levoketoconazole group and 14 (64%) of 22 patients in the prior placebo group, representing 58% normalization overall. Of 12 patients in the levoketoconazole group with normal mUFC at restoration phase baseline, 7 (58%) had normal mUFC at the end of the restoration phase. Of 9 patients in the levoketoconazole group with above-normal mUFC at restoration phase baseline, 4 (44%) had normal mUFC at the end of the restoration phase. One patient in the placebo group had normal mUFC at the end of the RW phase, and one additional patient had mUFC normalized by the restoration phase baseline assessment. mUFC remained normal in both patients at the end of the restoration phase. Of 20 prior placebo group patients with above-normal mUFC at restoration baseline, 12 (60%) had normal mUFC at the end of the restoration phase. Median time from the start of the restoration phase to first normalization for the placebo group was 25 days. Mean decreases in mUFC from restoration phase baseline (− 88.7 nmol/24 h in the levoketoconazole group and − 396.6 nmol/24 h in the placebo group) resulted in comparable mean values at the end of the restoration phase (135.6 nmol/24 h and 141.3 nmol, respectively). Similarly, mean increases in total and LDL-cholesterol observed in the placebo group during the RW phase were reversed during the restoration phase (Online Resource 4).

### Safety

In the safety population for all study phases combined, mean (SD) duration of exposure to levoketoconazole was 160.1 (78.4) days, with a mean (SD) daily dose of 538.7 (213.1) mg. In the ITT population, mean duration of exposure to study medication in the RW phase was 50.5 days for levoketoconazole (mean daily dose, 717.9 mg) and 28.6 days for placebo.

#### Adverse events

During the RW phase, the incidence of AEs was similar in the levoketoconazole and placebo groups (Table 3). The only AE reported for more than 2 patients in a treatment group during the RW phase was hypertension, which was reported for 3 patients in the levoketoconazole group and 1 patient in the placebo group; each event represented worsening of pre-existing hypertension. AEs occurring in 2 patients in a treatment group were headache (2 patients in each treatment group), nausea, and fatigue (each reported for 2 patients in the levoketoconazole group and 1 in the placebo group). AEs occurring more frequently with placebo were dizziness and insomnia (2 patients each versus none with levoketoconazole).

**Table 3:** Summary of adverse events

	Randomized withdrawal phase		Levoketoconazole All phases combined (n = 84)
Adverse events, n (%)	Levoketoconazole (n = 22)	Placebo (n = 22)	
Any AE	11 (50.0)	10 (45.5)	75 (89.3)
Drug-related AE^a^	2 (9.1)	0 (0)	34 (40.5)
Serious AE	1 (4.5)	0 (0)	13 (15.5)
AE leading to discontinuation	0 (0)	0 (0)	15 (17.9)
Severe AE	2 (9.1)	1 (4.5)	19 (22.6)
AEs of special interest^b^	1 (4.5)	0 (0)	23 (27.4)
Liver-related	0 (0)	0 (0)	9 (10.7)
QT prolongation	0 (0)	0 (0)	9 (10.7)
Adrenal insufficiency	1 (4.5)	0 (0)	8 (9.5)

*AE* adverse event, *MedDRA* Medical Dictionary for Regulatory Activities

^a^AEs that were assessed by the investigator as probably or definitely related to levoketoconazole ^b^Reported by the investigator as an AE of special interest; may not include all recorded AEs for the associated MedDRA preferred terms

During treatment with levoketoconazole across all study phases (n = 84), 75 (89%) patients had at least one treatment-emergent AE. The most common AEs (incidence ≥ 10%) were nausea (29%), hypokalemia (26%), hypertension (24%), headache (23%), diarrhea (14%), QT prolongation (14%), dizziness (13%), decreased appetite (12%), fatigue (12%), and vomiting (11%). Most AEs were of mild or moderate intensity; the most common severe AEs were abdominal pain, hypertension, hypokalemia, and nausea (3 patients each).

Per investigator judgment, 15 (18%) of 84 patients, all in the TM phase, discontinued levoketoconazole because of an AE. Serious AEs were reported among 13 (16%) patients, with 4 patients reporting at least 1 event considered probably or definitely related to levoketoconazole: 3 liver-related, 1 abdominal pain, 1 hypokalemia. During treatment with levoketoconazole, 32 AEs of special interest were reported among 23 (27%) patients: liver-related (9 patients [10.7%]), QT prolongation (9 patients [10.7%]), and adrenal insufficiency (8 patients [9.5%]). All AEs leading to discontinuation, serious AEs, and severe AEs reported during treatment with levoketoconazole are listed in Online Resource 5.

#### Liver tests

ALT values above the ULN occurred at least once among 37 (45%) of 83 patients with at least 1 postbaseline measurement during treatment with levoketoconazole, including 6 patients with ALT between 3 × and 5 × ULN and 3 patients with ALT above 5 × ULN (1 between 5 × ULN and 10 × ULN and 2 between 10 × ULN and 20 × ULN; Table 4). AST elevations of potentially clinically significant magnitude occurred only in the context of elevated ALT.

**Table 4:** Liver test monitoring, worst observed values

	Levoketoconazole All phases combined (n = 83)^a^	RW phase		Restoration phase	
		Levoketoconazole (n = 22)	Placebo (n = 22)	Levoketoconazole (n = 21)	Placebo (n = 22)
ALT					
> ULN	37 (44.6)	5 (22.7)	0 (0)	4 (19.0)	1 (4.5)
> 3 × to 5 × ULN	6 (7.2)	0 (0)	0 (0)	0 (0)	0 (0)
> 5 × ULN	3 (3.6)	0 (0)	0 (0)	0 (0)	0 (0)
AST					
> ULN	24 (28.9)	3 (13.6)	0 (0)	2 (9.5)	1 (4.5)
> 3 × to 5 × ULN	2 (2.4)	0 (0)	0 (0)	0 (0)	0 (0)
> 5 × ULN	2 (2.4)	0 (0)	0 (0)	0 (0)	0 (0)
GGT					
> ULN	32 (38.6)	6 (27.3)	1 (4.5)	6 (28.6)	1 (4.5)
> 3 × to 5 × ULN	6 (7.2)	0 (0)	0 (0)	0 (0)	0 (0)
> 5 × ULN	6 (7.2)	0 (0)	0 (0)	0 (0)	0 (0)
Total bilirubin					
> ULN	3 (3.6)	1 (4.5)	0 (0)	0 (0)	1 (4.5)
> 1.5 × ULN	0 (0)	0 (0)	0 (0)	0 (0)	0 (0)

*ALT* alanine aminotransferase, *AST* aspartate aminotransferase, *GGT* gamma glutamyl transferase, *RW* randomized withdrawal, *ULN* upper limit of normal

^a^One patient had missing post-baseline values

Across all study phases, 25 unique treatment-emergent AEs related to liver test abnormalities were reported for 19 (23%) patients. There were no cases meeting Hy’s Law criteria,^2^ and all cases of liver test abnormalities associated with AEs demonstrated reversibility without clinical sequelae, usually within 4 weeks of the first elevation above 3 × ULN. In 7 patients, levoketoconazole was permanently discontinued as a direct result of the liver abnormality. The recognized onset of potentially clinically significant liver test abnormalities was always during the TM phase, with the latest first onset occurring on Day 126; median time to first ALT greater than 3 × ULN among the 9 affected patients was 61 days.

#### Electrocardiogram

In the overall safety population, 17 (20.2%) of 84 patients had at least 1 Fridericia-corrected QT (QTcF) interval representing an increase of more than 60 ms from baseline at any time during treatment with levoketoconazole. Three (3.6%) patients had a worst-recorded absolute QTcF interval between 481 and 500 ms, and 3 (3.6%) had intervals greater than 500 ms. There was no evidence of clinical sequelae relating to any QTcF prolongation event. There were no AEs related to ventricular arrhythmias, nor other symptoms directly associated with QT prolongation events.

#### Adrenocorticotropic hormone concentration and tumor size

Mean ACTH concentration in the subset of 62 patients diagnosed with CD was 13.9 pmol/L (1.3 × ULN) at TM baseline. Using the highest observed value per patient during the TM phase, mean ACTH increased by 12.8 pmol/L. At baseline of the RW phase, mean ACTH concentration was 30.3 pmol/L (2.8 × ULN) in the levoketoconazole group (n = 18) and 27.1 pmol/L (2.5 × ULN) in the placebo group (n = 20). At the end of the RW phase, mean change in ACTH concentration was 4.3 pmol/L in the levoketoconazole group and − 12.9 pmol/L in the placebo group (treatment difference, 17.3; *P* = 0.1019). Mean change in ACTH concentration from restoration phase baseline at the end of the restoration phase was 10.0 pmol/L and − 6.1 pmol/L in the prior placebo and levoketoconazole groups, respectively. Pituitary MRI scans were available at RW phase baseline (last scan prior to the first dose of the study medication in the RW phase) for 37 of 38 patients diagnosed with CD. Of patients with a visible tumor (n = 18), 1 in each group had a macroadenoma and 16 (9 levoketoconazole and 7 placebo) had a microadenoma. Follow-up MRI scans at the end of the RW phase (approximately 31–60 days after the RW baseline assessment) were available for 16 patients, 8 of whom had visible tumors. Overall mean change in tumor diameter was − 0.5 mm with levoketoconazole (n = 4) and 0.5 mm with placebo (n = 4), with a maximum increase of 1 mm in each group. There were no reported treatment-emergent AEs of Nelson’s syndrome, pituitary apoplexy, or pituitary enlargement during study.

#### Testosterone

Among female patients with TM phase testosterone data, mean total (n = 56) and free (n = 55) testosterone concentrations at TM baseline were in the upper half of the normal ranges; among male patients with data (n = 14), mean free testosterone concentration was at the low end of the normal range and mean total testosterone was below normal (Online Resource 6). Mean total and free testosterone levels decreased for both male and female patients: to the lower half of the reference ranges at the last TM phase observation for females, and below the lower limit of normal for males. For both male and female patients, increases in mean total and free testosterone observed in the placebo group during the RW phase were reversed at the end of the restoration phase (Online Resource 6).

#### Body weight, body mass index, and vital signs

At TM baseline, mean body weight and body mass index (BMI) were, on average, indicative of a mildly obese population (Online Resource 7). Mean decreases in body weight and BMI were observed at the last observation in the TM phase. During the RW phase, mean changes in body weight and BMI were increased in the placebo group and decreased in the levoketoconazole group. During the restoration phase, further mean decreases in body weight and BMI were observed in the levoketoconazole group; mean values in the placebo group were essentially unchanged from restoration phase baseline (Online Resource 7). There were no notable findings related to mean changes in vital signs, including blood pressure, during the study (Online Resource 8).

## Discussion

LOGICS is the first placebo-controlled study investigating the efficacy of levoketoconazole in the treatment of patients with endogenous CS. The enrolled population was generally representative of adults with CS who require drug treatment. Primary results from LOGICS demonstrate a difference between continuing masked active treatment versus switching to matching placebo following establishment of mUFC control (loss of response in 95.5% of the placebo group vs. 40.9% of the levoketoconazole group; treatment difference, − 54.5%; *P* = 0.0002). Furthermore, restoration of active therapy was largely successful in reversing therapy withdrawal to placebo, with 60% of placebo-treated patients with elevated mUFC following placebo treatment showing normal mUFC at study completion. Therefore, the effect of levoketoconazole to normalize mUFC is shown to be drug-specific, reversible upon drug withdrawal, and restorable upon drug reintroduction, confirming and extending findings of the completed SONICS study [24]. We have also shown that restoration of normal cortisol secretion can be achieved much more quickly than initial dose finding when titrating to a previously established effective dose, with a median time to normalization of 3 to 4 weeks using the rapid titration schedule (increments of 150 mg/day every third day) in the restoration phase.

The mUFC normalization in the LOGICS study appears to be at least partly a reflection of the baseline mUFC, with higher pretherapy mUFC portending the need for higher doses of levoketoconazole to normalize mUFC and, perhaps, also a lower likelihood of cortisol secretion normalization. Notwithstanding this observation, all studied doses demonstrated effectiveness in at least 1 patient each, and mUFC was normalized at the last TM assessment in 4 of the 6 patients with the highest recorded baseline mUFC in LOGICS (range: about 14- to 40-fold ULN).

The dosing findings further establish that empirical dose-finding on a per-patient basis is effective and, as of this writing, remains the only known way to determine a therapeutic dose. However, losses of mUFC response (loss of normalization) during continued active treatment in the RW phase of LOGICS were common—occurring in approximately 40% of patients. Therefore, sustained mUFC normalization should not be assumed to result from an initial mUFC normalization demonstration—a minority of patients will lose mUFC control in the short-term and will never regain it with levoketoconazole monotherapy, while others can recover a state of mUFC normalization, or otherwise acceptable UFC control, through maintenance of dose or one or more subsequent dose increases (as also shown during maintenance in SONICS) [24]. Routine cortisol monitoring beyond the initial mUFC normalization is universally advisable to maintain UFC at goal, with dose increases as needed up to the maximal studied dose or as tolerated. In addition, due to the high degree of variability associated with UFC assessments, more-reliable parameters of efficacy are needed to better monitor treatment outcomes [11].

LDL-cholesterol serves as an important biomarker of cardiovascular risk, and cardiovascular disease is the primary correlate of excess mortality in CS [5, 33, 34]. Open-label levoketoconazole was associated with reduction of mean LDL-cholesterol at the end of the TM phase in the current study, an effect that rapidly reversed upon withdrawal in the placebo group but not in the continued levoketoconazole group. These findings establish that the effect of levoketoconazole to reduce total and LDL-cholesterol during open-label treatment is confirmed as a treatment-related effect.

The safety profile observed during the LOGICS study was consistent with prior studies; no new safety signals or unexpected findings of clinical relevance were observed from the AE, ECG, or laboratory data. Nausea, hypokalemia, headache, and hypertension were the most commonly reported AEs during levoketoconazole treatment across study phases. There is little evidence that any common AE was dose-related.

Hypokalemia AEs were reported more frequently in LOGICS compared with SONICS despite a similar incidence of measured serum potassium below the normal range [24]. The more frequent reporting of mild potassium abnormalities such as hypokalemia AEs was likely explained by the study team encouraging investigators to maintain potassium concentration in the upper half of the normal range (i.e., between 4.5 and 5.3 mmol/L) to mitigate the risk of potassium-related prolongation of the QT interval.

The incidence of suspected or confirmed adrenal insufficiency of about 10% was somewhat higher than previously observed, which may relate to the higher average daily levoketoconazole dosage used during dose titration in this study [24]. All adrenal insufficiency cases were considered to be mild or moderate in severity, and only 1 patient discontinued participation as a result. As with other medical therapies for CS, however, clinicians must adjust levoketoconazole dosing to balance optimal cortisol control against the risk of adrenal insufficiency.

The randomized withdrawal to placebo provided an opportunity to observe AEs associated with acute treatment withdrawal versus active therapy maintenance. However, the limited duration of the placebo comparison meant that only frequent and early-onset events of this type would be expected to be observed at higher rates in the placebo arm. Only 2 AE terms—insomnia and dizziness—were reported in at least 2 more patients in the placebo arm relative to the levoketoconazole arm. Insomnia and dizziness are frequently reported by patients with CS, both before and after cortisol withdrawal [35]. Therefore, their appearance at a nominally higher frequency during placebo treatment is inconclusive.

Mean increases in QTc interval were established in this study as being at least partially drug-related and also likely dose-related, consistent with the putative mechanism of KCNH2 I_Kr_ channel inhibition [36, 37]. However, only 3 patients experienced interval prolongation above the clinically important threshold of 500 ms, and there was no evidence of clinical sequelae (e.g., arrhythmia) relating to any QTcF prolongation event. Most patients experiencing a QT interval prolongation event were able to remain in the study at the same or reduced dosage following dose interruption.

Liver test monitoring findings essentially mirrored prior experience [24], with commonly seen abnormalities of transaminases (AST, ALT, and gamma glutamyl transferase) above the ULN. Abnormalities above the threshold of 3 × ULN (incidence about 11% for ALT) always first occurred during titration, with a median onset about 2 months after initiating treatment, which could be important for monitoring algorithms. There were no patients whose data met Hy’s Law, which portends a higher risk of serious liver injury; indeed, the highest recorded total bilirubin concentration was 1.1 × ULN. Most potentially clinically significant cases of liver test abnormalities were asymptomatic, emphasizing the importance of monitoring. All liver test abnormalities were documented as reversible, and there were no clinical sequelae, highlighting success of the monitoring and risk mitigation scheme employed.

Common laboratory changes during the TM phase of LOGICS were mostly those expected based on the pharmacological properties of levoketoconazole to reduce cortisol and androgen concentrations in blood [23, 24]. Reduction of total and free testosterone, a pharmacological effect presumably caused by potent levoketoconazole inhibition of cytochrome P450 17A (CYP17A) activity [21, 23], was observed in both male and female patients, with salutary effects on hirsutism and acne likely a result. As testosterone reduction in men in particular is potentially an adverse effect, testosterone should be monitored occasionally, or as prompted by symptoms suggestive of hypogonadism during use of levoketoconazole in men.

Limitations of this study include the short duration of the RW phase, particularly in patients assigned to placebo who (with 1 exception) required early rescue treatment. The RW phase design necessarily selects a population that has tolerated the medication prior to randomization, in this case for at least 14 weeks, confounding interpretation of safety findings post-randomization. Furthermore, the relatively small sample size of the ITT population led to some baseline differences between groups, although these differences were not found to have meaningfully affected interpretation of the results. It was necessary to change the statistical plan because the intended primary analysis (logistic regression model) inflated the apparent treatment effect of levoketoconazole, and the prespecified supportive analysis is considered a more accurate estimate of treatment efficacy. Data for levoketoconazole are limited to comparisons versus placebo, as there are no head-to-head comparisons with different drugs. Finally, strict inclusion and exclusion criteria and a study population largely of European descent limited patient heterogeneity.

In conclusion, treatment with levoketoconazole benefitted many patients in the current study, as evidenced by frequent normalization of mUFC and concurrent improvements in lipid profile. These benefits were established as related specifically to treatment with levoketoconazole via loss of benefit upon withdrawal to placebo and restoration upon reintroduction of active therapy. Additional novel findings are the relationship of dose to both UFC normalization and QTc prolongation and that restoration of levoketoconazole following an interruption not due to an AE may be safely accomplished by rapid re-titration. Levoketoconazole was generally well tolerated and had a safety profile that was manageable with appropriate close monitoring. Together these findings support the use of levoketoconazole as a treatment option for adult patients with CS.

## Supplementary Information

Below is the link to the electronic supplementary material.

Supplementary file1 (PDF 301 kb)

## Data Availability

Only the summarized data provided in the manuscript and supplemental online resource items is to be shared.

## Footnotes

1 As per protocol, patients who completed SONICS (through 12 months of maintenance treatment) within 6 months of LOGICS screening visit and demonstrated maintenance of clinical benefit on a stable therapeutic dose of levoketoconazole for at least 12 weeks immediately prior to screening were eligible to enter LOGICS. SONICS completers who did not fulfill these requirements were potentially eligible if they met the inclusion criteria; however, the criteria pertaining to elevated mUFC and LNSC (or abnormal dexamethasone suppression test) did not apply for patients currently using levoketoconazole.

2 Hy’s Law components: (1) higher incidence of ≥ threefold elevations above the ULN of ALT or AST for study drug versus placebo, (2) ≥ 1 patient with aminotransferase elevation(s) also shows elevation of serum total bilirubin to > 2 × ULN, and (3) no other reason can be found for the combination of increased aminotransferase(s) and total bilirubin (e.g., viral hepatitis, preexisting or acute liver disease) [32].
