# Supplementary material for: Levoketoconazole in the treatment of patients with endogenous Cushing’s syndrome: a double-blind, placebo-controlled, randomized withdrawal study (LOGICS)
Source: Pituitary. 2022 Sep 9;25(6):911–26. doi: 10.1007/s11102-022-01263-7 (PMC9675660; doi:10.1007/s11102-022-01263-7)
Supplement: Supplementary file 1 — Supplementary file1 (PDF 301 kb) [file 11102_2022_1263_MOESM1_ESM.pdf]

Online Resource 1 Kaplan-Meier curve for time from randomized withdrawal phase baseline to loss of therapeutic response during the randomized withdrawal phase (ITT population)

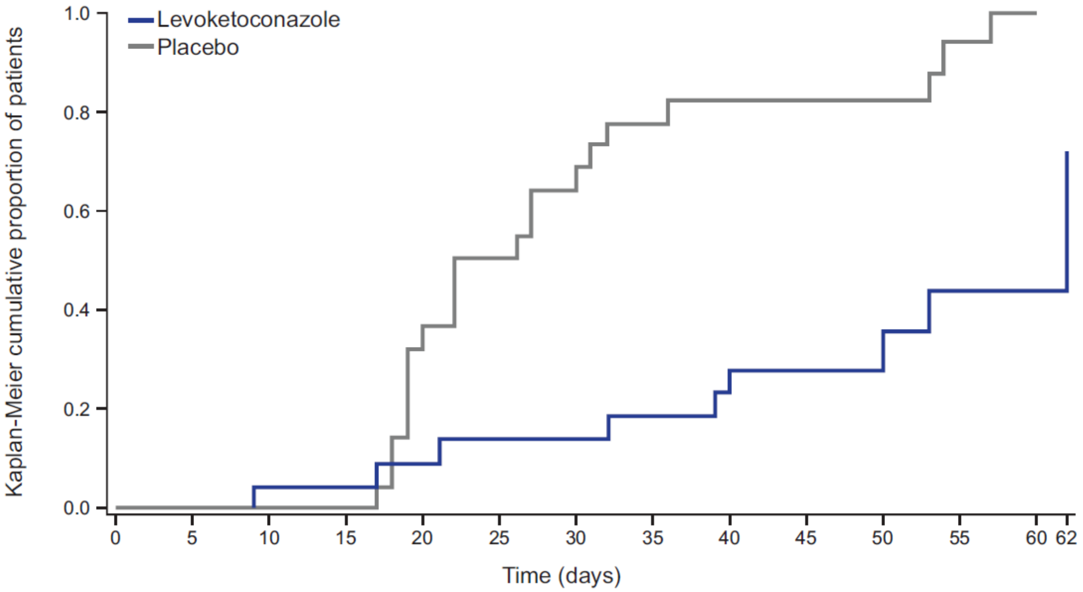

Patients at risk	0	5	10	15	20	25	30	35	40	45	50	55	60	62
Levoketoconazole	22	22	21	21	20	19	19	18	16	15	9	6	2	2
Placebo	22	22	22	22	15	11	8	5	4	4	4	1	0	0

Online Resource 2 Normalization of mUFC during the titration-maintenance phase

	Last Levoketoconazole Dose Level During the TM Phase (mg/day)								
	150	300	450	600	750	900	1050	1200	Overall
Patients by dose, n	2	7	14	18	16	8	7	7	79
Patients with mUFC normalization									
At any time during TM, n (%)	1 (50.0)	5 (71.4)	11 (78.6)	14 (77.8)	15 (93.8)	7 (87.5)	5 (71.4)	2 (28.6)	60 (75.9)
At the end of TM, n (%)	0	4 (57.1)	10 (71.4)	10 (55.6)	9 (56.3)	5 (62.5)	3 (42.9)	1 (14.3)	42 (53.2)<br>95% CI[b]<br>(41.6, 64.5)
Patients with mUFC normalization or partial response[a]									
At any time during TM, n (%)	1 (50.0)	5 (71.4)	12 (85.7)	17 (94.4)	15 (93.8)	8 (100)	6 (85.7)	5 (71.4)	69 (87.3)
At the end of TM, n (%)	0	5 (71.4)	12 (85.7)	13 (72.2)	11 (68.8)	8 (100)	5 (71.4)	3 (42.9)	57 (72.2)<br>95% CI[b]<br>(60.9, 81.7)
Patients with mUFC normalization, excluding patients who discontinued (non-safety and/or non-efficacy reasons)[c]									
Patients by dose, n	7		10	13	13	8	5	7	63
At any time during TM, n (%)	5 (71.4)		9 (90.0)	12 (92.3)	13 (100)	7 (87.5)	3 (60.0)	2 (28.6)	51 (81.0)
At the end of TM, n (%)	4 (57.1)		9 (90.0)	8 (61.5)	8 (61.5)	5 (62.5)	2 (40.0)	1 (14.3)	37 (58.7)<br>95% CI[b]<br>(45.6, 71.0)

mUFC mean urinary free cortisol, *TM* titration maintenance

[a]Partial response is defined as having at least 50% decrease in mUFC from the TM phase baseline

[b]95% CI is calculated based on Fisher's exact test. If a patient had a missing mUFC at their last dose level but had a nonmissing mUFC at their penultimate dose level and did not discontinue the phase due to lack of efficacy, then this nonmissing mUFC would be used in the analysis. All other patients were considered as not having achieved mUFC normalization at the end of the TM phase

[c]Patients who discontinued the TM phase because of withdrawal of consent, sponsor decision, protocol deviation, or physician decision are excluded

Online Resource 3 Mean changes in other secondary outcome measures in the titration-maintenance and randomized withdrawal phases

	TM phase Levoketoconazole (n = 79)		RW phase Baseline mean (SD)		Change from RW baseline at the end of the RW phase Mean (SD)[a]			
	Baseline mean (SD)	Mean (SD) change	Levoketoconazole (n = 22)	Placebo (n = 22)	Levoketoconazole (n = 22)	Placebo (n = 22)	Treatment Difference[b]	Adjusted *P* value[c]
CushingQoL, total score[d]	42.5 (20.9)	4.6 (10.8)	54.1 (20.3)	46.4 (18.7)	0.7 (9.6)	–2.3 (11.5)	3.0 (3.2)	0.7210
BDI-II, total score[e]	17.8 (12.7)	–3.1 (7.2)	12.3 (10.4)	15.4 (8.7)	–0.8 (4.8)	–0.2 (8.7)	–0.5 (2.2)	0.8092
Acne, global score[f]	1.7 (5.7)	–0.5 (4.1)	1.7 (3.1)	1.0 (3.4)	–0.2 (1.3)	1.0 (4.1)	–1.1 (0.9)	0.2229
Hirsutism (females), total score[g]	6.2 (7.5)	–1.2 (4.5)	5.4 (5.9)	5.3 (4.8)	–2.9 (5.6)	0.0 (0.9)	–2.9 (1.5)	0.1496
Peripheral edema, total score[h]	1.5 (3.0)	0.1 (2.3)	1.0 (1.9)	0.9 (1.9)	–0.1 (0.5)	0.5 (1.5)	–0.6 (0.3)	0.1496

BDI-II Beck Depression Inventory II, *CS* Cushing's syndrome, *QoL* quality of life, *RW* randomized withdrawal, *SD* standard deviation, *TM* titration maintenance

Sample sizes vary slightly across specific measurements due to data availability

[a]These are considered exploratory analyses because some biomarkers of CS comorbidities (the previous set in the hierarchical analysis plan) failed to demonstrate the superiority of levoketoconazole over placebo

[b]All values shown are mean (SD) except for treatment difference, which is mean (standard error)

[c]Treatment comparison at the end of the RW phase by two-sample t-test with Hochberg adjustment

[d]CushingQoL score could range from 0 (worst) to 100 (best)

[e]BDI-II total score could range from 0 (best) to 63 (worst), with depression severity considered minimal for a score of 0–13, mild for 14–19, moderate for 20–28, and severe for 29–63

[f]Acne global score could range from 0–44, for which 0 indicated none, 1–18 mild, 19–30 moderate, 31–38 severe, and ≥39 very severe
[g]Hirsutism total score could range from 0 (none) to 36 (worst).
[h]Peripheral edema total score could range from 0 (none) to 12 (worst)

Online Resource 4 Mean change in mUFC and biomarkers of CS comorbidities during the restoration phase (ITT population)

	RES phase baseline mean (SD)		End of RES phase mean (SD)		Mean (SD) change from RES baseline at the end of RES phase		Mean (SD) change from RW baseline at the end of RES phase	
	Levoketoconazole (n = 21)	Placebo (n = 22)	Levoketoconazole (n = 21)	Placebo (n = 22)	Levoketoconazole (n = 21)	Placebo (n = 22)	Levoketoconazole (n = 21)	Placebo (n = 22)
mUFC (nmol/24h)	224.3 (341.3)	537.9 (346.0)	135.6 (87.3)	141.3 (130.3)	–88.7 (359.7)	–396.6 (301.5)	55.0 (93.0)	52.8 (128.8)
LDL-cholesterol (mmol/L)	1.9 (0.7)	2.7 (0.8)	2.1 (0.5)	2.1 (0.9)	0.1 (0.6)	–0.7 (0.5)	0.00 (0.4)	0.02 (0.4)
Total cholesterol (mmol/L)	4.0 (0.9)	5.2 (0.8)	4.2 (0.7)	4.4 (0.9)	0.2 (0.7)	–0.9 (0.6)	0.05 (0.5)	0.10 (0.5)
FBG (mmol/L)	5.0 (0.5)	5.3 (1.0)	5.3 (1.4)	5.4 (1.5)	0.2 (1.2)	0.1 (1.0)	0.07 (1.0)	0.2 (0.7)
HbA1c (%)	5.6 (0.6)	5.7 (0.8)	5.7 (1.0)	5.8 (0.8)	0.08 (0.51)	0.11 (0.22)	0.03 (0.4)	0.2 (0.3)
hsCRP (mg/L)	4.1 (6.0)	1.6 (2.0)	2.2 (3.4)	6.9 (16.6)	–2.0 (3.5)	5.3 (16.9)	–0.6 (3.6)	0.5 (20.5)
Fasting insulin (pmol/L)	112.3 (69.5)[a]	109.1 (55.9)[a]	102.7 (51.7)	100.3 (70.5)	–9.7 (35.2)	–8.9 (49.2)	–27.0 (99.9)	0.00 (63.0)
HOMA-IR	4.2 (3.0)[a]	4.3 (2.7)[a]	3.8 (1.9)	4.0 (3.5)	–0.4 (1.7)	–0.4 (2.4)	–1.3 (4.8)	–0.06 (3.1)

CS Cushing's syndrome, *FBG* fasting blood glucose, *HbA1c* hemoglobin A1c, *HOMA-IR* homeostatic model assessment of insulin resistance, *hsCRP* high-sensitivity C-reactive protein, *ITT* intent-to-treat, *LDL* low-density lipoprotein, *mUFC* mean urinary free cortisol, *RES* restoration, *RW* randomized withdrawal, *SD* standard deviation, *ULN* upper limit of normal

Reference ranges: mUFC: ULN = 138 nmol/24 h; LDL-cholesterol: 0–3.35 mmol/L; total cholesterol: 0–5.15 mmol/L; FBG: 3.9–6.4 mmol/L (13–49 years old), 3.9–6.9 mmol/L (≥50 years old); HbA1c: 0–6.5%; hsCRP: 0–3 mg/L; fasting insulin: 30–90 pmol/L; HOMA-IR: reference range not established.

[a]Levoketoconazole: n = 18; placebo: n = 21

Online Resource 5 Adverse events leading to discontinuation, serious adverse events, and severe adverse events during treatment with levoketoconazole (safety population)

Levoketoconazole (n = 84)					
AEs leading to discontinuation, n (%)		Serious AEs, n (%)		Severe AEs, n (%)	
Nausea	3 (3.6)	Hypokalemia	3 (3.6)	Hypokalemia	4 (4.8)
Decreased appetite	2 (2.4)	Abdominal pain	2 (2.4)	Abdominal pain	3 (3.6)
Liver disorder	2 (2.4)	Liver disorder	2 (2.4)	Hypertension	3 (3.6)
QT prolonged	2 (2.4)	Drug-induced liver injury	1 (1.2)	Nausea	3 (3.6)
Vomiting	2 (2.4)	Epistaxis	1 (1.2)	Liver disorder	2 (2.4)
Abdominal pain	1 (1.2)	Fatigue	1 (1.2)	ALT increased	1 (1.2)
Adrenal insufficiency	1 (1.2)	Hepatic enzyme increased	1 (1.2)	Asthenia	1 (1.2)
ALT increased	1 (1.2)	Limb injury	1 (1.2)	Bone pain	1 (1.2)
Bone pain	1 (1.2)	Myocardial infarction	1 (1.2)	Bronchitis	1 (1.2)
Cold sweat	1 (1.2)	Nausea	1 (1.2)	Decreased appetite	1 (1.2)
Drug-induced liver injury	1 (1.2)	Nephropathy, toxic	1 (1.2)	Drug-induced liver injury	1 (1.2)
Headache	1 (1.2)	Pancreatitis, chronic	1 (1.2)	Fatigue	1 (1.2)
Hepatic enzyme increased	1 (1.2)	Umbilical hernia	1 (1.2)	Hepatic enzyme increased	1 (1.2)
Hepatic function abnormal	1 (1.2)	Upper respiratory tract infection	1 (1.2)	Hyperamylasemia	1 (1.2)
Hypersensitivity	1 (1.2)	Urosepsis	1 (1.2)	Hyperlipasemia	1 (1.2)
Hypertension	1 (1.2)	Vomiting	1 (1.2)	Muscular weakness	1 (1.2)
Liver injury	1 (1.2)			Myocardial infarction	1 (1.2)
Myocardial infarction	1 (1.2)			Nephropathy, toxic	1 (1.2)
Rash	1 (1.2)			Pancreatitis, chronic	1 (1.2)
				Tooth abscess	1 (1.2)
				Umbilical hernia	1 (1.2)
				Urosepsis	1 (1.2)
				Vomiting	1 (1.2)

AE adverse event, *ALT* alanine aminotransferase

Online Resource 6 Mean changes in testosterone concentrations

	TM phase Levoketoconazole (n = 79)		RW phase Baseline mean (SD)		Change from RW baseline at the end of the RW phase Mean (SD)			
	Baseline mean (SD)	Mean (SD) change[a]	Levoketoconazole (n = 22)	Placebo (n = 22)	Levoketoconazole (n = 22)	Placebo (n = 22)	Treatment Difference[b]	Adjusted P value[c]
Males	n = 14	n = 14	n = 7	n = 3	n = 7	n = 3		
Total testosterone (nmol/L)	8.53 (5.84)	–2.82 (4.32)	8.49 (3.36)	3.46 (3.98)	–1.05 (4.98)	2.29 (1.67)	–3.34 (2.11)	0.1536
Free testosterone (nmol/L)	0.16 (0.10)	–0.08 (0.08)	0.13 (0.06)	0.07 (0.10)	–0.03 (0.08)	0.08 (0.08)	–0.11 (0.05)	0.0971
Females	n = 56	n = 56	n = 15	n = 19	n = 14	n = 19		
Total testosterone (nmol/L)	0.80 (0.54)	–0.30 (0.60)	0.30 (0.14)	0.34 (0.30)	0.00 (0.16)	0.45 (0.53)	–0.45 (0.13)	0.0019
Free testosterone (nmol/L)	0.013 (0.012)[d]	–0.007 (0.013)[d]	0.003 (0.002)	0.003 (0.004)	0.001 (0.003)	0.011 (0.020)[e]	–0.010 (0.005)	0.0494

	RES phase baseline mean (SD)		End of RES phase mean (SD)		Mean (SD) change from RES baseline at the end of RES phase	
	Levoketoconazole (n = 21)	Placebo (n = 22)	Levoketoconazole (n = 21)	Placebo (n = 22)	Levoketoconazole (n = 21)	Placebo (n = 22)
Males	n = 7	n = 3	n = 7	n = 3	n = 7	n = 3
Total testosterone (nmol/L)	7.44 (6.40)	5.75 (5.40)	7.43 (5.27)	4.62 (4.41)	–0.01 (4.55)	–1.13 (2.43)
Free testosterone (nmol/L)	0.095 (0.085)	0.16 (0.17)	0.067 (0.051)[f]	0.092 (0.101)	–0.003 (0.07)[f]	–0.064 (0.090)
Females	n = 14	n = 19	n = 12	n = 18	n = 12	n = 18
Total testosterone (nmol/L)	0.31 (0.19)	0.79 (0.53)	0.28 (0.16)	0.38 (0.32)	–0.05 (0.17)	–0.44 (0.55)

Free testosterone (nmol/L)	0.004 (0.004)	0.014 (0.02)	0.003 (0.002)	0.004 (0.004)	–0.001 (0.004)	–0.010 (0.021)

RES restoration. *RW* randomized withdrawal, *SD* standard deviation, *TM* titration maintenance

Reference ranges: total testosterone: females: 0.07–1.56 nmol/L (18–69 years old), 0.07–1.39 nmol/L (70–94 years old); males 8.68–38.17 nmol/L (18–69 years old; no males ≥70 years old in the study); free testosterone: females: 0.0003–0.0222 (18–69 years old), 0.0007–0.0128 nmol/L (70–89 years old); males: 0.1215–0.5379 nmol/L (18–69 years old; no males ≥70 years old in the study)

[a]Last observed value

[b]Values shown are mean (SD) except for treatment difference, which is mean (standard error)

[c]Treatment comparison at the end of the RW phase by two-sample t-test

[d]n = 55

[e]n = 18

[f]n = 6

Online Resource 7 Mean changes in body weight and BMI

	TM phase Levoketoconazole (n = 79)[a]		RW phase baseline Mean (SD)		Change from RW baseline at the worst post-baseline result during RW phase Mean (SD)			
	Baseline mean (SD)	Mean (SD) change[b]	Levoketoconazole (n = 22)	Placebo (n = 22)	Levoketoconazole (n = 22)	Placebo (n = 22)	Treatment Difference[c]	Adjusted *P* value[d]
Body weight (kg)	83.7 (18.5)	–3.0 (3.9)	81.5 (22.6)	79.3 (12.8)	–0.7 (1.9)	1.8 (2.2)	–2.4 (0.6)	0.0003
BMI (kg/m^2)	31.4 (6.8)	–1.1 (1.5)	30.6 (8.4)	29.8 (5.0)	–0.2 (0.07)	0.6 (1.0)	–0.8 (0.3)	0.0027

	RES phase baseline mean (SD)		End of RES phase mean (SD)		Change from RES baseline at the end of RES phase mean (SD)	
	Levoketoconazole (n = 21)	Placebo (n = 22)	Levoketoconazole (n = 21)	Placebo (n = 22)	Levoketoconazole (n = 21)	Placebo (n = 22)
Body weight (kg)	80.1 (22.4)	81.1 (13.0)	78.2 (22.6)[e]	81.1 (13.7)	–1.8 (3.2)[e]	0 (2.7)
BMI (kg/m^2)	30.0 (8.3)	30.4 (5.0)	29.3 (8.5)[e]	28.8 (8.2)	–0.6 (1.2)[e]	0.0 (1.1)[f]

BMI body mass index, *DBP* diastolic blood pressure, *RES* restoration, *RW* randomized withdrawal, *SBP* systolic blood pressure, *SD* standard deviation, *TM* titration maintenance

BMI categories: underweight, <18.5 kg/m^2; normal, 18.5 to <25 kg/m^2; overweight, 25 to <30 kg/m^2; obese, ≥30 kg/m^2

[a]n = 72

[b]Last observed value

[c]Values shown are mean (SD) except for treatment difference, which is mean (standard error)

[d]Treatment comparison at the end of the RW phase by two-sample t-test

[e]n = 20

[f]One outlier was excluded

Online Resource 8 Mean changes in blood pressure

	TM phase Levoketoconazole (n = 79)[a]		RW phase baseline mean (SD)		Change from RW baseline at the worst post-baseline result during RW phase mean (SD)			
	Baseline mean (SD)	Mean (SD) change[b]	Levoketoconazole (n = 22)	Placebo (n = 22)	Levoketoconazole (n = 22)	Placebo (n = 22)	Treatment Difference[c]	Adjusted *P* value[d]
SBP (mm Hg)	131.9 (13.5)	–0.4 (18.5)	133.3 (14.8)	130.1 (12.7)	–0.9 (19.0)	2.3 (16.4)	–3.2 (5.3)	0.5549
DBP (mm Hg)	84.2 (10.0)	–0.7 (11.8)	85.1 (8.8)	83.5 (10.2)	–3.4 (8.9)	0.4 (8.6)	–3.8 (2.6)	0.1592

	RES phase baseline mean (SD)		End of RES phase mean (SD)		Change from RES baseline at the end of RES phase mean (SD)	
	Levoketoconazole (n = 21)	Placebo (n = 22)	Levoketoconazole (n = 21)	Placebo (n = 22)	Levoketoconazole (n = 21)	Placebo (n = 22)
SBP (mm Hg)	132.0 (18.4)	132.4 (15.8)	133.3 (20.7)	129.6 (12.9)	1.3 (13.8)	–2.9 (14.8)
DBP (mm Hg)	81.1 (7.7)	83.9 (12.0)	83.4 (11.9)	81.8 (10.2)	2.3 (8.2)	–2.2 (9.3)

DBP diastolic blood pressure, *RES* restoration, *RW* randomized withdrawal, *SBP* systolic blood pressure, *SD* standard deviation, *TM* titration maintenance

Blood pressure categories: SBP: low, <90 mmHg; normal, 90–139 mmHg; high, 140–169 mmHg; very high: >169 mmHg; DBP: low, <50 mmHg; normal, 50–89 mmHg; high, 90–109 mmHg; very high, >109 mmHg

[a]n = 77

[b]Last observed value

[c]Values shown are mean (SD) except for treatment difference, which is mean (standard error)

[d]Treatment comparison at the end of the RW phase by two-sample t-test
